# Benchmarking functional brain network organization in childhood and its similarity to adults

**DOI:** 10.3389/fnimg.2026.1818662

**Published:** 2026-04-28

**Authors:** Sana A. Ali, Damion V. Demeter, Abigail R. Baim, Emily M. Koithan, Matthew Feigelis, Salma Zreik, Jonathan Ahern, Sarah E. Chang, Sujin Park, Evan M. Gordon, Scott Marek, Deanna J. Greene

**Affiliations:** 1 Department of Cognitive Science, University of California, San Diego, La Jolla, CA, United States; 2 Mallinckrodt Institute of Radiology, Washington University School of Medicine, St. Louis, MO, United States

**Keywords:** brain networks, development, fMRI, functional connectivity, resting state

## Abstract

Understanding how large-scale functional networks mature across development is essential for linking brain organization to cognition and behavior. Thus, the population-level organization of functional networks in children and how it compares to adult network architecture deserves further study. Using resting-state fMRI data from 7,316 children aged 9–10 years in the Adolescent Brain Cognitive Development (ABCD) Study, we mapped functional networks in matched discovery (*n* = 3,624) and replication (*n* = 3,692) cohorts in the cerebral cortex, basal ganglia, thalamus, and cerebellum. Functional connectivity and network topography were highly reproducible across child cohorts, demonstrating that large-scale network organization can be reliably estimated in childhood at the population scale. Comparisons with the adult Human Connectome Project (HCP; *n* = 1,000) dataset revealed reduced cross-age similarity compared to the within-age similarity. Our findings indicate that by late childhood, the global scaffold of brain networks approximates adult architecture with continued refinement, particularly in higher-order association systems. This large-scale, discovery–replication framework establishes a reproducible benchmark for cross-age functional network mapping, providing a foundation for longitudinal analyses of maturation across adolescence.

## 1 Introduction

Large-scale, functional brain networks provide the organizational framework for complex human cognition and behavior (Fox et al., 2005; Petersen and Sporns, 2015). These networks can be measured at rest using functional connectivity (FC) MRI, which leverages temporally coherent fluctuations in activity across distributed brain regions to capture the brain’s intrinsic architecture (Biswal et al., 1995). This network architecture is highly structured, consisting of distinct functional systems that support sensorimotor, cognitive, and affective processes (Power et al., 2011a; Thomas Yeo et al., 2011; Gordon et al., 2016). The modular organization of these systems is characterized by strong within-network coupling and systematic between-network segregation, forming the basis of higher-order association areas (Sporns and Betzel, 2016; Bassett and Sporns, 2017; Braga and Buckner, 2017). Although whole brain functional network organization has been well characterized in adults, both in group average data (Thomas Yeo et al., 2011; Greene et al., 2014a; Uddin et al., 2019) and in densely-sampled individuals (Laumann et al., 2015; Gordon et al., 2017; Marek et al., 2018), questions remain regarding how functional networks mature throughout childhood and adolescence (Power et al., 2012; Grayson et al., 2014; Marek et al., 2015a).

During infancy and early childhood, functional networks emerge from localized patterns of connectivity that gradually integrate into distributed systems supporting flexible cognition (Fransson et al., 2007; Gao et al., 2009; Smyser et al., 2010; Damaraju et al., 2014; Wylie et al., 2014; De Asis-Cruz et al., 2015; Van Den Heuvel et al., 2015). Studies in children have shown that somatomotor and visual networks exhibit adult-like features early in life (Gao et al., 2009, 2011, 2013b), whereas control and attention networks show more protracted maturation throughout adolescence (Fair et al., 2013; Gao et al., 2013a; Gu et al., 2015; Marek et al., 2015a). These findings suggest that while the general organization of functional networks are detectable in childhood, properties related to segregation, integration, and network-to-network interactions continue to evolve across development (Satterthwaite et al., 2013; Sporns, 2013; Grayson et al., 2014; Gu et al., 2015; Grayson and Fair, 2017; Marek et al., 2019a; Tu et al., 2025b). Yet the extent to which children’s organization and connectivity resembles adult patterns remains incomplete.

Several methodological factors pose challenges for studying whole-brain functional network organization in developmental samples. Pediatric studies are often prone to motion-related artifacts and variable data quality, complicating efforts to assess reproducibility and specificity of observed effects (Power et al., 2012; Greene et al., 2016, 2018; Dosenbach et al., 2017). In addition, estimating reliable FC patterns in subcortical structures presents distinct challenges, including reduced temporal signal-to-noise ratio, smaller anatomical size, partial volume effects, and sensitivity to preprocessing choices, particularly in studies with small sample sizes (Ji et al., 2019). As a result, many developmental studies of large-scale functional networks have focused primarily on cortical regions, leaving cortico-subcortical organization less systematically characterized. This gap is notable given extensive reciprocal connections between the cortex, basal ganglia, thalamus, and cerebellum through recurrent loops that support integrated processing (Alexander et al., 1986; Greene et al., 2014b, 2020; Hwang et al., 2017; Palesi et al., 2017b, 2017a; Hintzen et al., 2018; Badke D’Andrea et al., 2023). Population-level characterizations of functional network organization in childhood would therefore benefit from large samples, stringent motion control, and inclusion of both cortical and subcortical systems. Moreover, a population scale network map can also serve as a benchmark for interpreting results from smaller developmental samples.

Population-scale neuroimaging initiatives now make it possible to address many of these challenges. The Adolescent Brain Cognitive Development (ABCD) Study provides high-quality, longitudinal fMRI data from thousands of children starting at age 9–10 years, offering the power needed to define reliable brain network maps development (Volkow et al., 2018). Similarly, the Human Connectome Project (HCP) offers a large, well-characterized adult reference cohort, facilitating comparison of functional network organization across age groups (Van Essen et al., 2012, 2013).

The present study leverages the ABCD and HCP datasets to characterize large-scale functional brain networks in childhood (using the largest low-motion dataset possible to date) and to quantify its similarity to adult network architecture. Using a discovery-replication design in the ABCD baseline dataset, we computed cortical and subcortical pairwise functional connectivity and derived graph-theoretical, data-driven (Rosvall and Bergstrom, 2008) brain network maps. We employed several quantitative similarity metrics to compare child and adult organization, including Dice coefficient, normalized mutual information (NMI), and Mantel label-based permutation tests, and assessed network integration using participation coefficient. Together, this work provides a population-scale benchmark of functional network organization in childhood and quantifies similarity to adults, while highlighting network-specific differences.

## 2 Materials and methods

### 2.1 Datasets

#### 2.1.1 ABCD dataset (child)

Resting-state functional connectivity (RSFC) data were obtained from the ABCD fast track portal (Feczko et al., 2021 BioRxiv). To create the final sample used in these analyses, children from the full baseline sample (*N* = 11,572) were divided into a discovery (*N* = 5,786) and replication (*N* = 5,786) samples (Table 1), matched across 10 demographic variables (site location, age, sex, ethnicity, grade, highest level of parental education, handedness, combined family income, and exposure to anesthesia) using the ABCD 2.0 data release. Family members (i.e., sibling pairs, twins, and triplets) were grouped in the same set, and the two sets were matched to include equal numbers of single participants and family members.

**Table 1:** Participant demographics.

Variable	Child discovery (*N* = 3,624)	Child replication (*N* = 3,692)	*p*-value
Sex	F = 1,831; M = 1,793	F = 1,818; M = 1,874	0.30^1^
Age mean	9.9 years (0.62)	9.9 years (0.62)	0.99^2^
Average frames included	1,140.7 (243.9)	1,142.6 (246.5)	0.65^2^
Handedness	R = 2,920, L = 257, A = 446; Missing data = 1	R = 2,971; L = 244; A = 71; Missing data = 6	<0.001^1^
Race and ethnicity			
White	2,022 (55.80%)	2,115 (57.29%)	0.34^1^
Black	466 (12.86%)	452 (12.24%)	0.54^1^
Hispanic	705 (19.45%)	644 (17.44%)	0.12^1^
Asian	67 (1.85%)	78 (2.11%)	0.23^1^
Other	361 (9.96%)	398 (10.78%)	0.27^1^
Not reported	3 (0.08%)	5 (0.14%)	0.48^1^

1 Chi-square.

2 Independent samples *t*-test.

R = Right-handed; L = Left-handed; A = Ambidextrous.

Strict motion exclusion criteria with regard to head motion were applied, given the systematic artifacts induced by motion in the scanner (Power et al., 2014). Specifically, participants with at least 600 frames (8 min) of low-motion (framewise displacement [FD] < 0.2 mm) RSFC data, with a minimum criteria of 5 contiguous frames required for data retention, were included. The final dataset consisted of RSFC data from a total of *N* = 7,316 youth ages 9–10 across the discovery (*N* = 3,624) and replication (*N* = 3,692) sets (Table 1).

Imaging data were collected across 21 U.S sites, harmonized across Siemens Prisma, Philips, and GE 3 T scanners, with 20 min of eyes-open (passive fixation) resting-state fMRI acquired using echo planar imaging (EPI) sequences (TR = 800 ms, TE = 30 ms, flip angle = 52°, field of view 216 × 216 mm, voxel size = 2.4 mm isotropic, 60 axial slices, multiband acceleration factor = 6). Full details on image acquisition can be found in Casey et al. (2018). Head motion was monitored during scans using the Framewise Integrated Real-time MRI Monitor (FIRMM) software at Siemens sites (Dosenbach et al., 2017).

#### 2.1.2 HCP dataset (adults)

We used publicly available high-resolution, preprocessed MRI data from the Human Connectome Project Adult S1200 (Van Essen et al., 2013) in this study. HCP MRI data were acquired on a Siemens Skyra 3 T scanner at Washington University in St. Louis, with approximately 15 min of eyes open (relaxed fixation) across 4 resting-state fMRI runs. HCP fMRI scanning included gradient-echo EPI sequences (TR = 720 ms, TE = 33.1 ms, flip angle = 52°, field of view 208 × 180 mm, voxel size = 2.0 mm isotropic, 72 axial slices, multiband acceleration factor = 8), collected with both left–right and right–left phase encoding. Full details on image acquisition can be found in Van Essen et al. (2013). We examined RSFC from 1,000 healthy adults (534 females; ages 22–37) who had four complete RSFC MRI runs with a FD < 0.2 mm.

### 2.2 Data processing overview

All data underwent preprocessing using the ABCD-HCP minimal pipeline (Sturgeon et al., 2021). For details on the full processing stream, see (Feczko et al., 2021) (BioRxiv). Briefly, the ABCD-HCP pipeline comprises six stages. (1) Normalization and alignment of anatomical data. (2) Parcellation and segmentation of the cortical surface from the normalized anatomical data using Freesurfer (Dale et al., 1999). (3) Surface outputs from Freesurfer are converted to CIFTIs, and volume data is transformed to a standard volume space (MNI) using ANTs’ nonlinear registration. (4) Volume data undergoes mean field distortion correction and resampling to 2-mm isotropic voxels in a single step using FSL’s applywarp tool. (5) The volumetric functional data is projected onto the surface. (6) The “DCANBOLDproc” step performs processing specific to functional connectivity data.

DCANBOLDproc steps include: First, a respiratory filter to improve FD estimates was calculated in the “vol” stage. Second, temporal masks were created to flag motion-contaminated frames using the improved FD estimates. These filtered FD values were used to compute temporal masks, with frames with FD > 0.3 mm being flagged at this stage to guide subsequent interpolation and nuisance regression. Following mask generation, the data are processed with the following steps: (1) demeaning and detrending, (2) interpolation across censored frames using least squares spectral estimation of the values at censored frames so that continuous data can be (3) general linear model “denoising” of data related to whole brain, ventricular, and white matter signals, as well as their derivatives. (4) A band-pass filter (0.008 Hz < *f* < 0.10 Hz) is then applied without re-introducing nuisance signals (Hallquist et al., 2013) or contaminating frames near high motion frames (Carp, 2013). (5) Respiratory motion filtering (Fair et al., 2020) and (6) motion censoring masks are created for a range of FD thresholds. All resting state data were then mapped to an MNI-transformed midthickness 32 k fs_LR surface mesh (Van Essen et al., 2012).

Following the DCANBOLDproc steps, the motion censoring masks were used to exclude frames exceeding a strict FD threshold of 0.2 mm and requiring retained data to include clusters of at least 5 contiguous, below FD threshold frames to minimize motion related artifacts and ensure meaningful functional signal. Spatial smoothing was then applied via geodesic Gaussian smoothing for surface data and volumetric smoothing for the subcortex and cerebellum (6 mm FWHM, 2.55 sigma) to create the final CIFTI dense time series file used for analyses.

### 2.3 Generation of cortical vertex-wise functional networks

For each participant in the ABCD discovery (*N* = 3,624) and replication (*N* = 3,692) cohorts, we computed pairwise correlations of BOLD time series for all cortical vertices. We then averaged the resulting correlations across all participants within each dataset, generating group-average cortical correlation matrices of dimensions 59,412 × 59,412. This group-average matrix was then used to identify cortical functional networks using the Infomap algorithm (Rosvall and Bergstrom, 2008), which is effective in detecting communities in a variety of complex networks, including social networks, biological networks, and technological networks (Yang et al., 2016). The group-average matrix underwent thresholding across a range of edge densities spanning from 0.1 to 5% and at each threshold, communities were identified using an adaptive version of the Infomap algorithm (Gordon et al., 2020). These communities were then assigned functional network identities through a template matching procedure, which compared each community to canonical, group-level network templates using the Jaccard index (Gordon et al., 2017) that assigned the best-matching network name if spatial overlap exceeded a predefined threshold (Jaccard > 0.15). To finalize network name assignments, a consensus network identity was selected for each vertex based on the sparsest threshold where a reliable match was found.

### 2.4 Subcortical network assignments in the ABCD dataset

Functional networks have been well characterized in the basal ganglia, thalamus, and cerebellum in both group and individual-level adult studies (Zhang et al., 2008; Buckner et al., 2011; Choi et al., 2012; Yuan et al., 2016; Hwang et al., 2017; Marek et al., 2018; Greene et al., 2020). To map functional networks in these structures, we implemented an approach previously used by our group and others. Specifically, voxels in these structures were assigned to functional networks based on their strongest functional connectivity with the cortical networks defined in the ABCD dataset in a winner-take-all fashion. Specifically, Pearson r correlations were computed between the time series from each subcortical voxel and the average time series across all vertices of each cortical functional network. Each voxel in the basal ganglia, thalamus, and cerebellum was then assigned to the cortical network with which it showed the highest correlation, enabling a straightforward extension of cortical network architecture into these regions. This approach yields a data-driven network organization scheme that reflects dominant functional affiliations, enabling the characterization of cortico-subcortical and cortico-cerebellar functional organization.

### 2.5 Identification of the Somato-Cognitive Action Network (SCAN)

In light of the recent discovery of the Somato-Cognitive Action Network (SCAN) as previously described by Gordon et al. (2023), we aimed to characterize the SCAN in the ABCD sample. We employed a manual seed-map approach by seeding inter-effector regions at a conservative 96–98% percentile connectivity threshold. Specifically, group-average functional connectivity was visually inspected using Connectome Workbench to locate candidate SCAN nodes showing strong within-network connectivity in the dense connectivity file, which contains vertex-wise correlation values across the cortex. To reduce subjectivity in localization, two raters (authors SA and SP) independently selected seed vertices for each inter-effector region. Seed-based SCAN masks were then generated per rater, compared, and reconciled to produce a consensus SCAN topography agreed upon by all raters. Guided by the SCAN topography described in Gordon et al. (2023), we identified three anchor nodes (top, middle, and bottom) that exhibited robust inter-node coupling. We then determined the raw connectivity thresholds (typically around ~98%) that best represented the spatial extent of each node.

### 2.6 Adult-child comparison

To enable comparison between child and adult cortical functional network organization, we pseudo-randomly selected a subset of 1,000 ABCD participants from each of the discovery and replication cohorts that were a sex-matched to the 1,000 HCP participants. Group-average vertex-wise correlation matrices were generated from the matched ABCD subset using the same preprocessing and Infomap steps described above. These matrices were then compared to the HCP group-average cortical correlation matrix. Due to the limited subcortical signal quality in the HCP dataset (Seitzman et al., 2020), adult-child comparisons were restricted to the cortex.

### 2.7 Quantifying similarities of functional network architecture

To assess reproducibility between child datasets and similarity between child and adult datasets, we used several complementary metrics to compare (1) vertexwise cortical FC, (2) functional network community structure, (3) functional network spatial topography, and (4) cross-network integration.

#### 2.7.1 Reproducibility of functional connectivity

To quantify reproducibility of large samples of children, we computed vertex-wise Pearson correlation coefficients (*r*) between the group-averaged functional connectivity matrices from the ABCD discovery and replication datasets. We repeated these analyses at the parcel-level using the 333 cortical parcels from (Gordon et al., 2016) to test at coarser, more easily visualized, granularity. This metric provides a global assessment of the similarity of large-scale connectivity patterns across two independent child cohorts.

#### 2.7.2 Functional network community structure

To compare network community structure, we computed the Normalized Mutual Information (NMI) between Infomap-derived network assignments from the ABCD discovery, replication, and HCP adult datasets. NMI is widely used for comparing clustering solutions and is well-suited for evaluating consistency in community detection results. NMI values range from 0 (no agreement) to 1 (perfect agreement). To assess statistical significance of similarity in cortical networks, we performed spatial permutation testing following the framework of Alexander-Bloch et al. (2018). Cortical network labels for the HCP dataset were rotated on a spherical surface (1,000 rotations). NMI was recomputed for each rotated map using the true ABCD labels to generate null distributions, and *p*-values (*p*_spin_) were calculated as the proportion of null values greater than or equal to the observed similarity.

#### 2.7.3 Functional network spatial correspondence

To assess how well specific network spatial topographies align or diverge between children and adults, we computed the Dice coefficient for each network, quantifying the spatial overlap between child and adult network maps. Dice values range from 0 to 1, where 0 indicates no overlap and 1 indicates perfect spatial alignment, providing network-specific differences in spatial organization across development. To assess whether network-specific dice overlap exceeded that expected given the spatial structure of cortical maps, we performed spin-based permutation testing for each network. For each comparison, one binary cortical network map was rotated on the spherical surface 1,000 times, and the Dice coefficient with the corresponding reference network was recomputed at each rotation to generate a null distribution. *p*_spin_ were calculated as the proportion of null Dice values greater than or equal to the observed Dice coefficient. Networks with *p* < 0.0045 (Bonferroni-corrected) were considered statistically significant.

To evaluate similarity in network-level connectivity patterns between children and adults, we used Mantel tests comparing the parcel-wise connectivity matrices from ABCD (discovery and replication) and HCP adults. Our goal was to test whether the observed correspondence exceeded the expected under a null distribution in which the mapping between parcels is randomized. We generate null matrices by permuting parcel labels before computing similarity, preserving the marginal distribution of connectivity strengths while disrupting systematic alignment across datasets. While this null does not fully account for spatial autocorrelation or network topology, it does provide a conservative baseline for assessing whether observed cross-age correspondence reflects more than random parcel-to-parcel alignment.

#### 2.7.4 Cross-network integration

To assess differences in how networks interact, we examined cross-network integration using the participation coefficient (PC). PC measures the extent to which a network node (here, a parcel) connects to multiple networks (Guimerà et al., 2007). PC is high when connections are distributed across many networks and low when they are concentrated within a single network. By comparing PC for each cortical parcel between child and adult datasets, we assess potential differences in the balance of segregation and integration that may reflect developmentally relevant organization of large-scale brain networks.

## 3 Results

### 3.1 Functional connectivity is highly replicable across child discovery and replication samples

**Figure 1:**
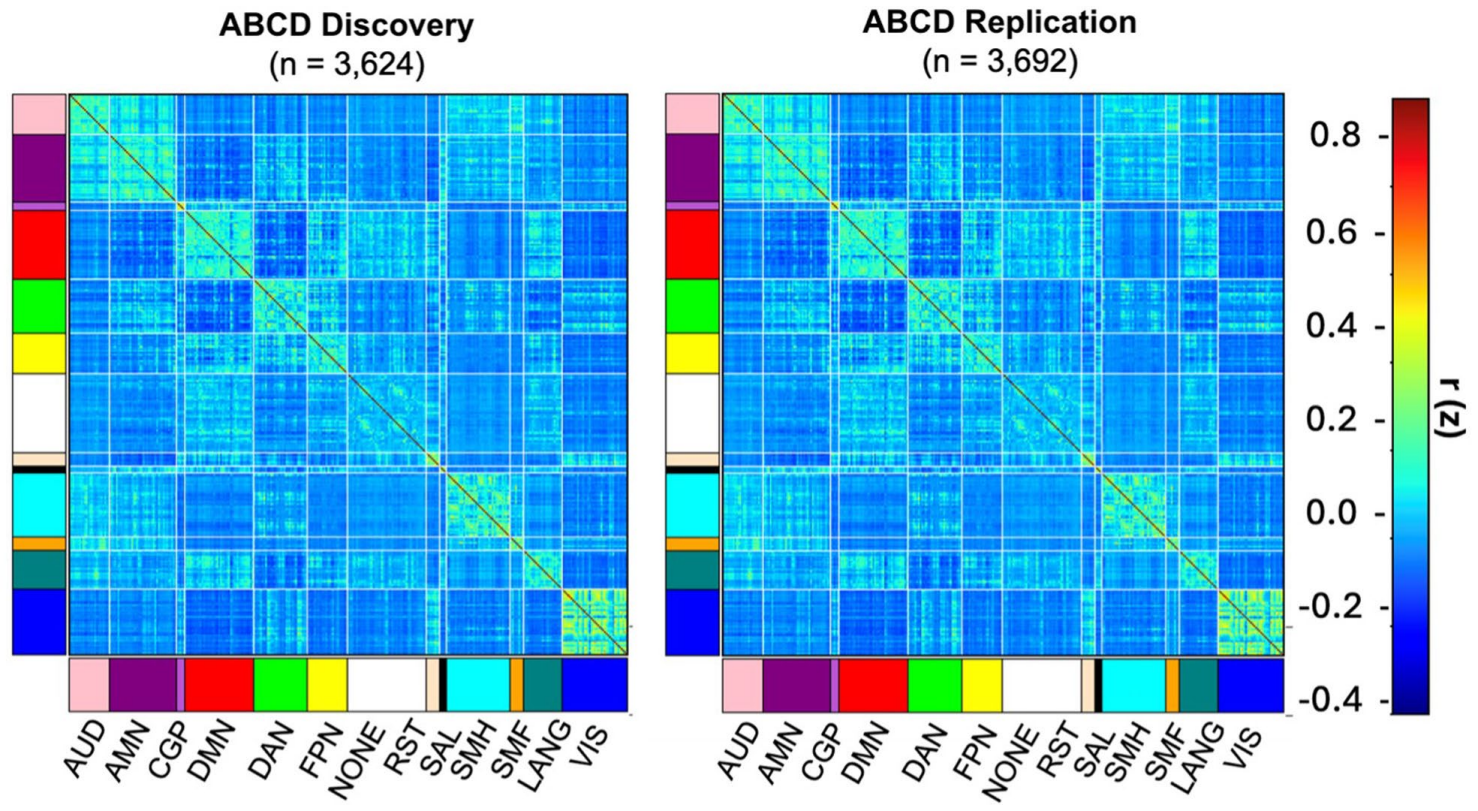
Parcel-wise group average correlation matrices across ABCD child discovery and ABCD child replication datasets (*r* = 0.99) visualized across canonical networks: auditory (AUD), AMN (action-mode network), cinguloparietal (CGP), default-mode (DMN), dorsal attention (DAN), frontoparietal (FPN), retrospinealtemporal (RST), salience (SAL), somatomotor hand (SMH), somatomotor face (SMF), language (LANG), visual (VIS).

We extended prior work (Marek et al., 2019b) demonstrating high reproducibility of functional connectivity in ABCD discovery and replication datasets, showing that this reproducibility scales to larger samples than previously examined. Extremely high similarity was found both at the vertex level (*r* = 0.99, *p* < 0.001) and at the parcel level (*r* = 0.99, *p* < 0.001). For visualization, Figure 1 displays the parcel level matrices.

### 3.2 Functional network topography is highly replicable across discovery and replication samples

**Figure 2:**
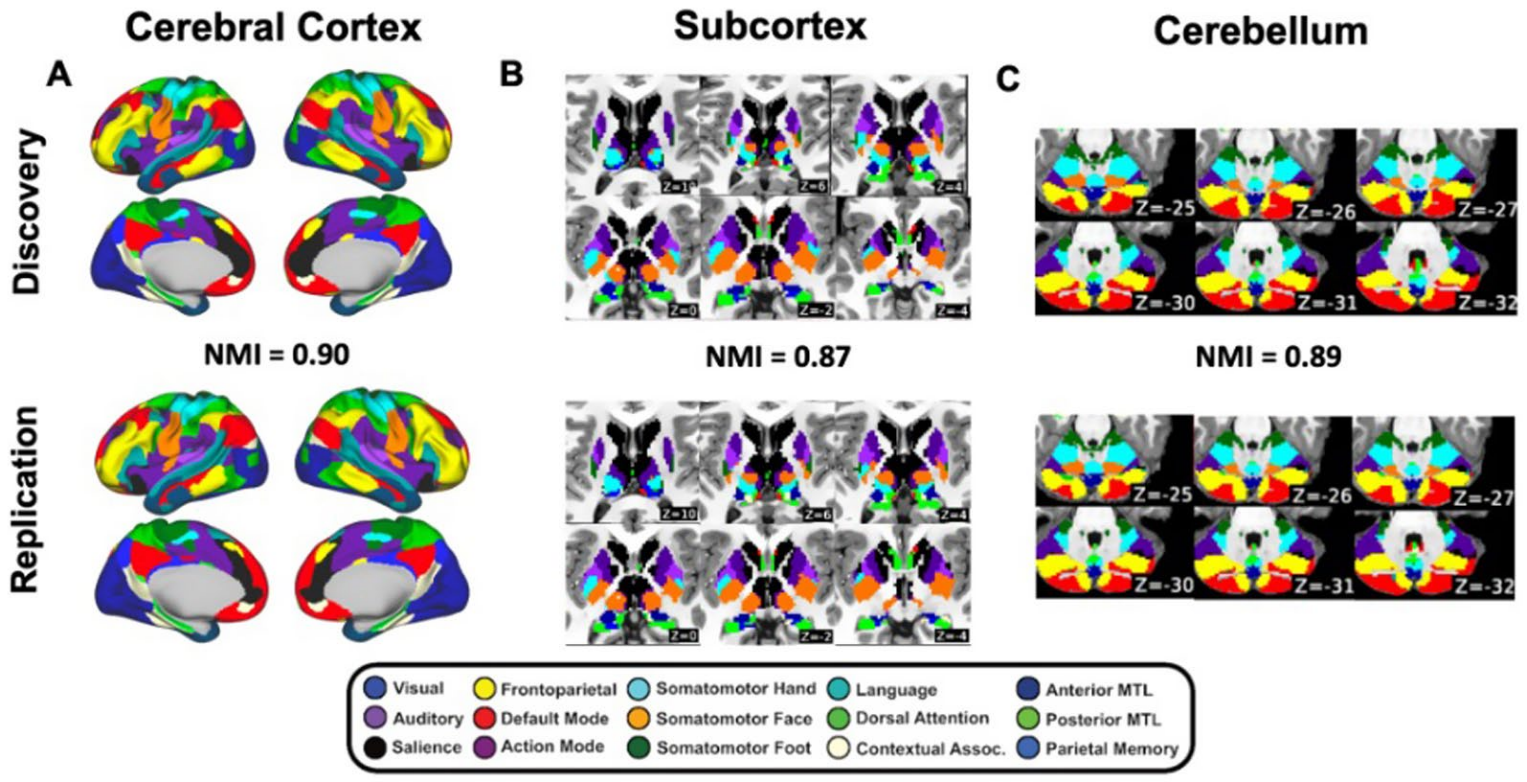
Group-average functional networks identified for the discovery (top) and replication (bottom) datasets in the **(A)** cerebral cortex (NMI = 0.90, *p*_spin_ < 0.001), **(B)** subcortex basal ganglia, thalamus; (NMI = 0.87, *p*_spin_ < 0.001), and **(C)** cerebellum (NMI = 0.89, *p*_spin_ < 0.001). Normalized mutual information. Colors denote defined functional network organization.

**Figure 3:**
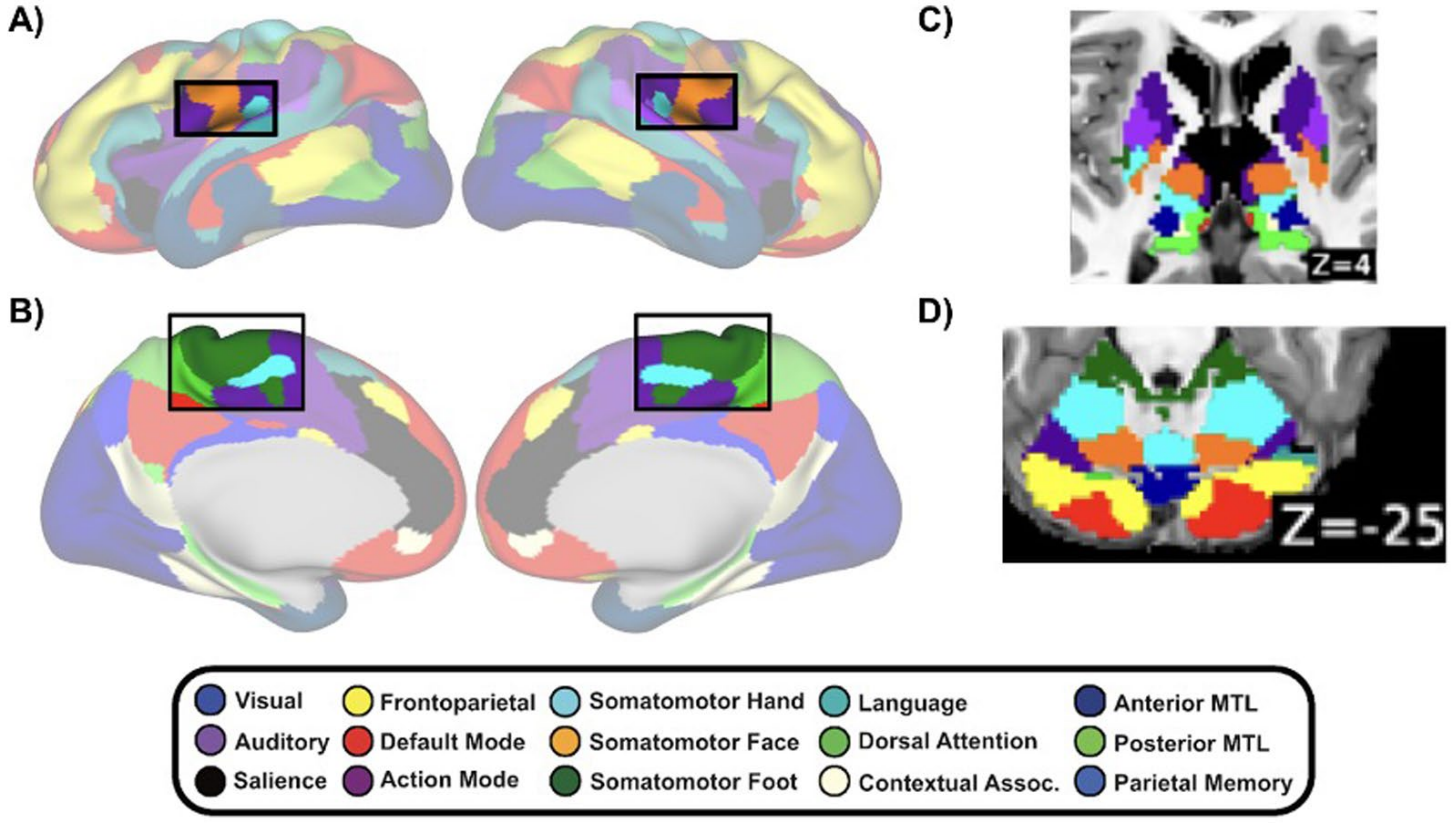
Replicable somatomotor topography across cortex, subcortical regions, and cerebellum. **(A,B)** Cortical somatomotor motifs on medial and lateral surfaces. **(C)** Subcortical somatomotor organization in the posterior putamen and central thalamus. **(D)** Cerebellar somatomotor representations. Patterns were independently observed in discovery and replication halves of the ABCD sample (*N* = 7,316). Colors denote defined functional network assignments.

We next identified large-scale functional networks in the cerebral cortex, basal ganglia, thalamus, and cerebellum separately in the discovery and replication sets (Figures 2A–C). In the cortex, there was highly similar functional network topography in the ABCD discovery and replication datasets, comprising canonical functional networks previously well-characterized in adults and in children, including the default-mode, frontoparietal, cingulo-opercular/action-mode, salience, dorsal attention, ventral attention, somatomotor, visual, and auditory networks. We identified the cortical networks with the highest functional connectivity to each subcortical and cerebellar voxel (Figures 2B,C), again resulting in qualitatively similar topography in these structures. Quantification of similarity with NMI between child discovery and replication datasets demonstrated that functional network assignments were highly reproducible in the cerebral cortex (NMI = 0.90, *p*_spin_ < 0.001; Figure 3A), basal ganglia and thalamus (NMI = 0.87, *p*_spin_ < 0.001; Figure 3B), and cerebellum (NMI = 0.89, *p*_spin_ < 0.001; Figure 3C).

The large sample size (*N* = 7,316) and the split-half design enabled robust confirmation of several previously described somatomotor motifs which were consistently evident across independent cohorts. On the medial wall of the cortex, we observed a somatomotor foot-hand-foot arrangement consistent with supplementary motor area (SMA) somatotopy (Figure 3B), a pattern noted previously (Penfield and Welch, 1951; Picard and Strick, 1996; Nachev et al., 2008) and here replicated in both cohorts. Moreover, the parietal operculum shows a somatomotor hand-face topography consistent with the known organization of secondary somatosensory cortex (SII) (Zilles et al., 1995; Eickhoff et al., 2007, 2010), again replicated across discovery and replication datasets (Figure 3A). In the subcortex, the posterior putamen and central thalamus display topographically organized somatomotor assignments in each split (Figure 3C). In the thalamus, the lateral geniculate nucleus (LGN) is also evident, consistent with expected visual relay anatomy. In the cerebellum, we observe the characteristic dorsal and ventral representation of the motor cortex reflecting the well-described doubled somatomotor topography of the motor cerebellum (Buckner et al., 2011). Together, these findings, which are robust and replicated across independent halves of the sample, highlight a stable, anatomically principled motor organization spanning cortical, subcortical, and cerebellar regions.

#### 3.2.1 SCAN at population scale in late childhood

**Figure 4:**
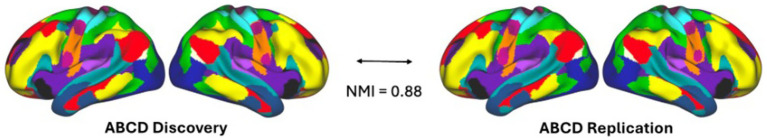
Group-average cortical functional networks identified for the discovery (left) and replication (right) datasets in 9–10-year-olds (NMI = 0.88, *p*_spin_ = 0.001). SCAN nodes are shown in burgundy. Colors denote defined functional network organization.

Building on the replicated somatomotor organization observed across cortex, subcortex, and cerebellum, we extended our analysis to include a newly discovered higher-order network that links motor effectors with control processes (Gordon et al., 2023). The SCAN has been previously identified in adult and pediatric group-average data, including the ABCD cohort (Gordon et al., 2023; Lynch et al., 2024). Here, leveraging the scale and reproducibility of our split half datasets we provide an additional population-level characterization of the SCAN in late childhood, situating it within the broader landscape of motor and control network organization described above, yielding a group-averaged map of SCAN organization (Figure 4).

Consistent with prior work, the SCAN was detectable at the group level and exhibited the characteristic distributed topography linking inter-effector regions (Figure 4). Relative to primary somatomotor networks, the SCAN appeared spatially distributed, consistent with its proposed role as an integrative system. The split half analysis (NMI = 0.88, *p*_spin_ = 0.001) provides novel evidence that the SCAN is a reproducible feature of large-scale functional organization in children. However, accurately delineating SCAN remains challenging, as existing automated approaches vary in their sensitivity to its ventral and integrative components. For example, alternative labeling strategies applied to Infomap solutions can recover aspects of SCAN but may not completely capture its full extent (Lynch et al., 2024). As more automated and validated approaches for identifying SCAN are developed, we can more confidently verify the location of this network in children.

### 3.3 Functional organization is similar between children and adults, but less similar than same-age children

**Figure 5:**
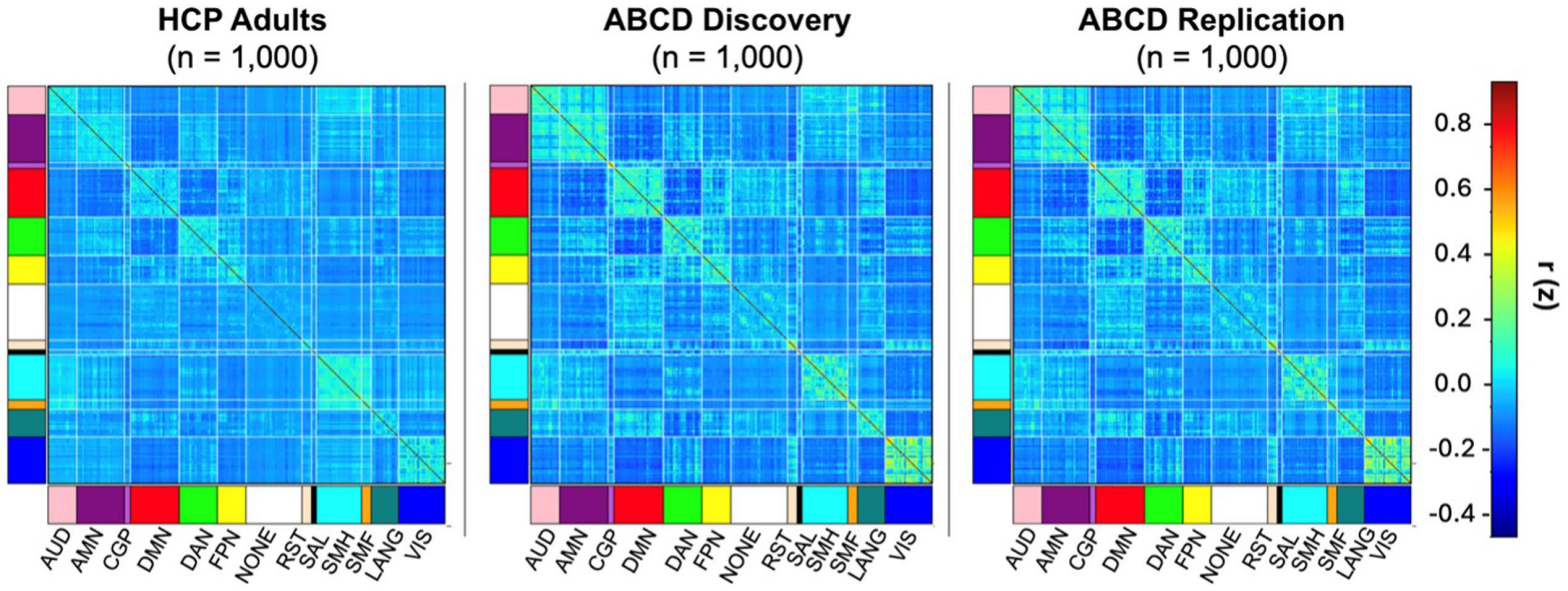
Parcel-wise group-average correlation matrices for HCP adults (left), ABCD discovery (middle), and ABCD replication (right) datasets visualized across canonical networks: auditory (AUD), AMN (action-mode network), cinguloparietal (CGP), default-mode (DMN), dorsal attention (DAN), frontoparietal (FPN), retrospinealtemporal (RST), salience (SAL), somatomotor hand (SMH), somatomotor face (SMF), language (LANG), visual (VIS).

To ensure comparable sample sizes, we subsampled the ABCD discovery and replication sets to *n* = 1,000 each, matched on sex, to parallel the adult HCP sample. Mantel tests comparing group-average parcel-wise connectivity matrices confirmed robust adult–child correspondence for both rank ordering (Spearman) and absolute values (Pearson) of functional connections (Figure 5). However, these adult–child similarities were consistently lower than the correspondence observed within the child cohort. Specifically, HCP vs. ABCD discovery yielded Spearman *r* = 0.8008, Pearson *r* = 0.8507 and HCP vs. ABCD replication yielded Spearman *r* = 0.8048, Pearson *r* = 0.8526 (all *p* < 0.0001 relative to the Mantel permutation null, generated by repeatedly permuting rows and columns of one matrix to obtain a chance distribution of correlations). In contrast, ABCD discovery vs. ABCD replication matrices showed near-identical agreement (Spearman *r* = 0.9980; Pearson *r* = 0.9988; *p* < 0.0001). Together, these findings indicate that by ages 9–10 years, whole brain functional network organization closely matches adult brain network architecture in both rank ordering and connection strength, and they reinforce the exceptional reproducibility of the child group averages. At the same time, this correspondence is lower than the extremely high similarity observed between the ABCD discovery and replication group averages.

**Figure 6:**
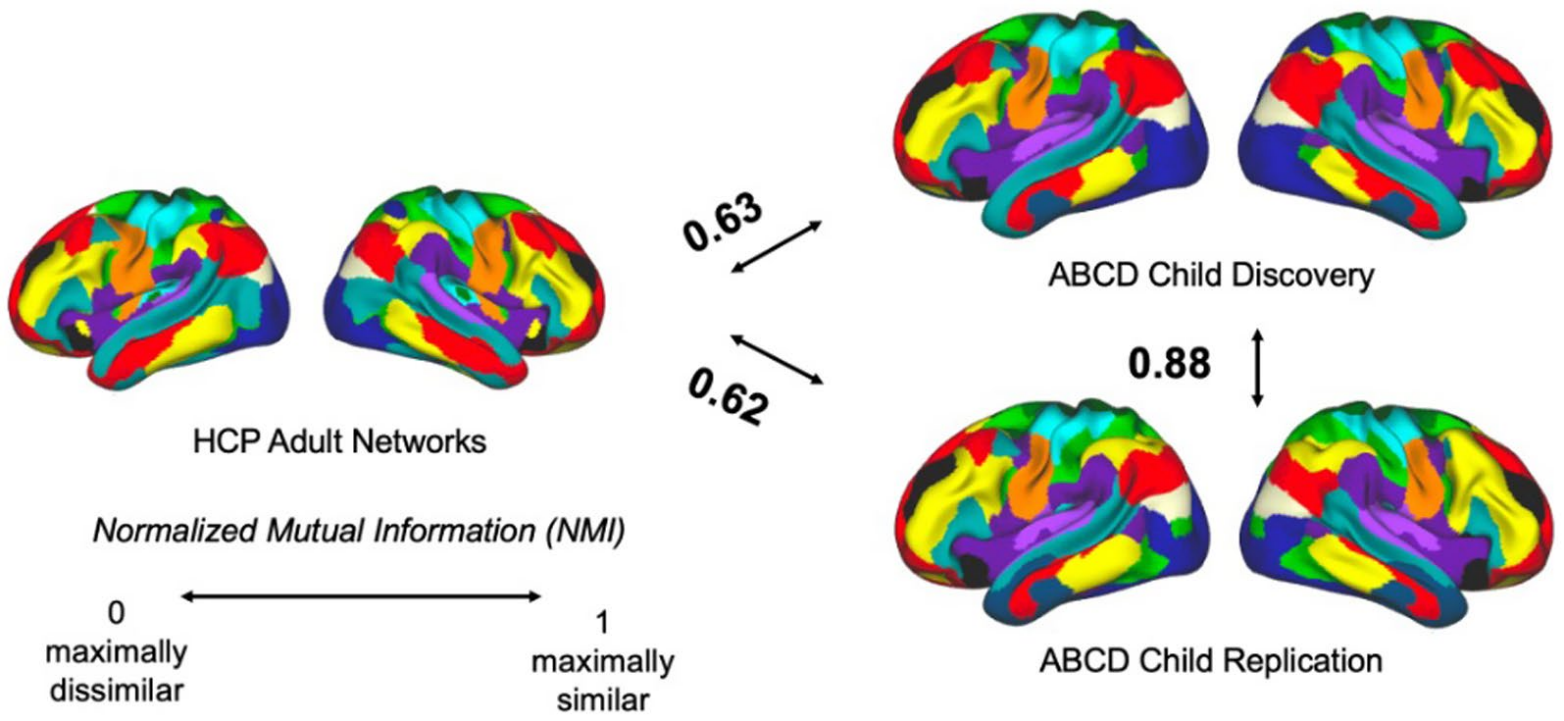
Similarity of functional network topography between age groups. NMI for child–child is higher than child-adult comparisons (Adult vs. Discovery: NMI = 0.63, *p*_spin_ = 0.001; Adult vs. Replication: NMI = 0.62, *p*_spin_ = 0.001). NMI: Normalized mutual information.

To compare cortical functional network topography in children and adults, we compared group-averaged functional network maps from the ABCD child cohorts to the HCP (*n* = 1,000) adult dataset (Van Essen et al., 2013). Quantification of similarity in the arrangement of large-scale functional networks (“community structure”) using NMI showed moderate adult-child similarity (NMI = 0.63 for adult-child discovery; 0.62 for adult-child replication). NMI between the two subsampled ABCD child partitions (*n* = 1,000 each) remained high (0.88; Figure 6). Spatial permutation testing confirmed that these values significantly exceeded those under spatially constrained null models (*p*_spin_ = 0.001 for both comparisons), indicating meaningful cross-age alignment of functional network topography.

**Figure 7:**
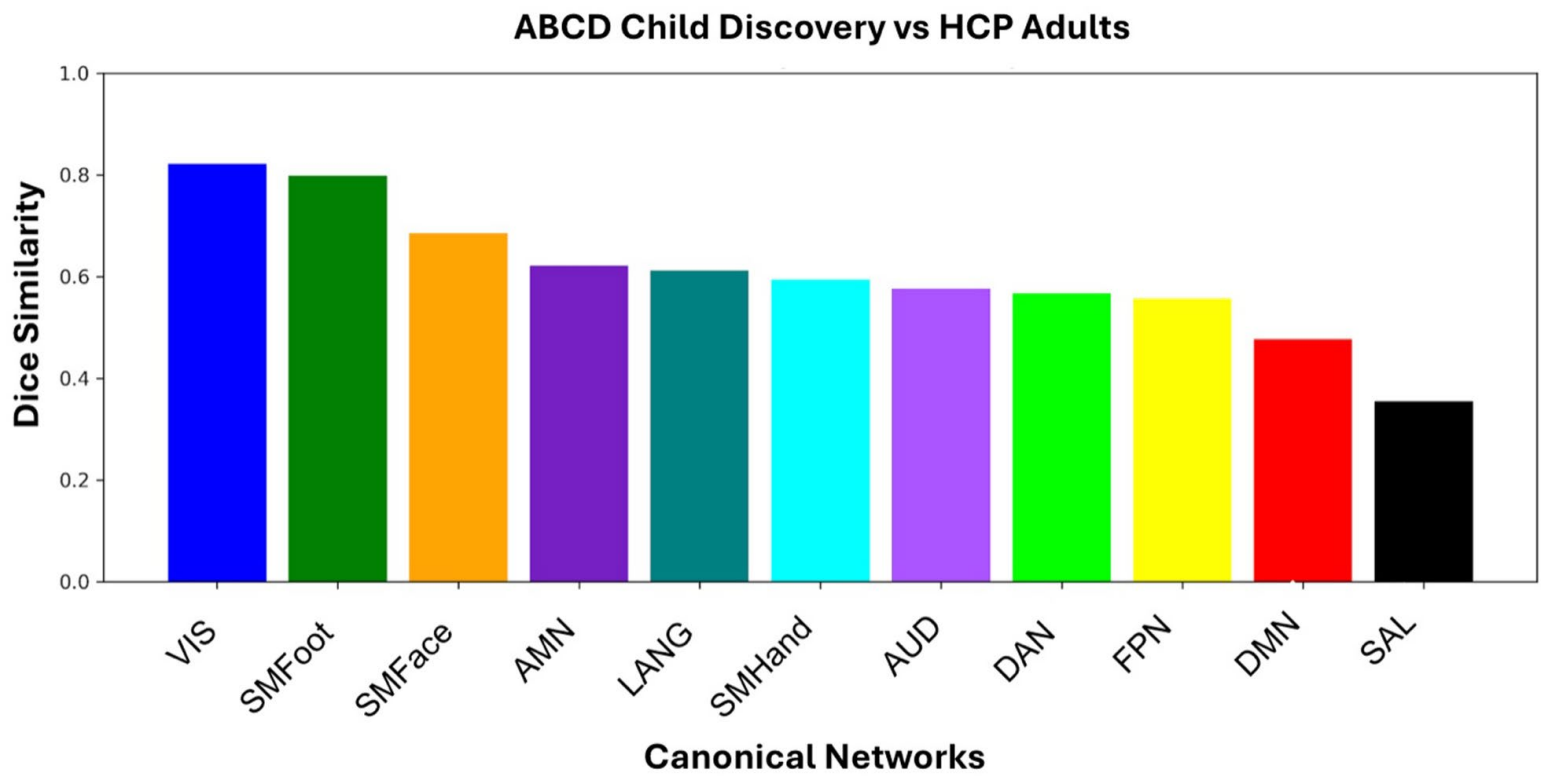
Dice overlap between the ABCD child discovery and HCP adult template across canonical networks: visual (VIS), somatomotor foot (SMFoot), somatomotor face (SMF), action-mode network (AMN), default-mode (DMN), language (LANG), frontoparietal (FPN), somatomotor hand (SMH), dorsal attention (DAN), salience (SAL). Significance of network-specific cortical Dice overlap was assessed using spin-based spatial permutation testing for 1,000 rotations (all *p*_spin_ ≤ 0.002).

To quantify spatial overlap and assess the similarity between children and adults in the topography of each functional network separately, we calculated spatial similarity with the Dice coefficient. Spin-based permutation testing showed that the observed child-adult Dice overlap exceeded spatially constrained null expectations for each network in both the discovery and replication cohorts (all *p*_spin_ ≤ 0.002). Figure 7 shows the magnitude of the overlaps with sensorimotor networks generally exhibiting higher overlap than association networks.

### 3.4 Cross-network integration

**Figure 8:**
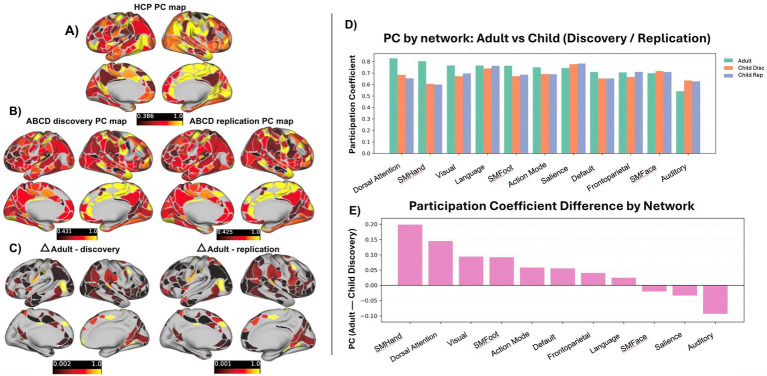
Participation coefficient (PC) across age groups. **(A)** Parcel-wise cortical PC in HCP adults. **(B)** Parcel-wise cortical PC in ABCD discovery and replication samples. Warmer colors indicate higher PC (as a measure of cross-network integration). **(C)** Adult–child difference maps (ΔPC = Adult − Child). **(D)** Network-wise mean PC values across groups. **(E)** Network-wise ΔPC (Adult − Child discovery).

To assess how functional network integration is distributed across large-scale systems, we evaluated cross-network connectivity using the PC, which indexes the extent to which a parcel’s connections span multiple functional networks. PC values were calculated on the group-average connectivity matrices using the Gordon-333 parcellation for the HCP cohort (Figure 8A), the ABCD discovery *n* = 1,000 cohort (Figure 8B left), and the ABCD replication *n* = 1,000 cohort (Figure 8B right). Parcels showing the largest difference in PC between children and adults were mostly overlapping across the two comparisons (HCP compared to each ABCD subsample; Figure 8C), indicating consistent patterns of developmental differences in cross-network connectivity at the parcel level. Figure 8D shows that compared to both ABCD discovery and replication samples, HCP adults had higher PC in dorsal attention, somatomotor-hand, somatomotor-foot, and visual networks. In contrast, children showed relatively higher PC in the auditory network, suggesting specificity in developmental differences in cross network interactions. Differences between age groups were smaller in control, default-mode, and somatomotor-face networks. Figure 8E displays the 𝚫PC between the HCP sample and ABCD discovery sample for each network to illustrate the magnitude of these effects.

## 4 Discussion

The present study provides a large-scale, population-level characterization of functional brain network organization in late childhood and quantifies its similarity with adults. Using the largest pediatric sample to date with low motion resting-state fMRI data and a demographically matched discovery-replication design (*N* = 7,316 total), we corroborated highly reproducible network organization across independent child cohorts (Marek et al., 2019a). The resulting networks exhibit strong biological plausibility, including well-defined canonical systems and anatomically principled motor organization spanning the cortex, subcortex, and cerebellum. Several of the organizational features observed here have been shown previously, but their stability at the population level has remained limited.

On the medial wall, we observed a foot-hand-foot arrangement corresponding with supplementary motor area somatotopy, aligning with classical descriptions of SMA body maps derived from stimulation, lesion, and neuroimaging studies (Penfield and Welch, 1951; Picard and Strick, 1996; Nachev et al., 2008). Similarly, the parietal operculum exhibited a hand-face organization consistent with the known topography of secondary somatosensory cortex (SII), aligning with cytoarchitectonic and functional imaging studies demonstrating somatotopic representations within SII (Zilles et al., 1995; Eickhoff et al., 2007, 2010). In subcortical regions, topographically organized somatomotor assignments within the posterior putamen and central thalamus were evident in both discovery and replication cohorts, consistent with known cortico-striatal and thalamo-cortical motor circuits supporting sensorimotor integration and action execution (Alexander et al., 1986; Haber, 2016). In the cerebellum, we observed the characteristic dorsal and ventral representations of motor cortex, reflecting the well-described doubled somatomotor topography of the motor cerebellum (Buckner et al., 2011). The consistent representation of these motifs across independent halves of a large pediatric sample confirms that these organizational features reflect stable properties of functional brain network organization.

Beyond canonical sensorimotor systems, the present study also demonstrates the population-level organization of the SCAN, a network recently described as interdigitated with effector specific motor regions and implicated in the integration of motor control with cognitive and goal-directed processes (Gordon et al., 2023). SCAN was originally identified using precision functional mapping approaches that leverage high-within subject reliability. Relying on group-averaged data and stringent thresholds may obscure individual representation, particularly due to the node size, and underestimate subtler, but potentially meaningful SCAN connectivity patterns. Future work incorporating subject-specific functional mapping will be crucial for better capturing the topography and variability of SCAN across development. Together, these findings demonstrate that both canonical functional systems and finer-grained somatomotor motifs are robustly and reproducibly expressed at the population level in children. By confirming the stability of these organizational features across independent cohorts, this study provides a reliable reference for large-scale functional network organization in childhood.

Across independent child cohorts, group-level RSFC and derived network organization were highly reproducible, indicating stable population-level architecture in late childhood. High correspondence between discovery and replication datasets suggests that the observed patterns are not driven by sample-specific effects or measurement noise, but instead reflect reliable features of large-scale functional organization. This reproducibility is particularly important in the context of developmental neuroimaging, where head motion and data quality can substantially influence estimates of functional connectivity. The robust within-age consistency observed here establishes a stable reference for interpreting future longitudinal analyses within the ABCD Study. As additional waves of imaging data become available, this population-level benchmark will facilitate the distinction between true within-subject change and variability attributable to measurement or analytic factors.

Our findings indicate that macroscopic human brain functional organization at 9–10 years old is substantially similar to adult network architecture. Prior work has shown that core features of network organization even emerge early in infancy and early childhood (Gao et al., 2011). Building on this foundation, the present study quantified the similarity of child and adult network architecture, demonstrating broad correspondence in functional connectivity and network topography. Nonetheless, not all systems are equivalent in their degree of alignment: sensorimotor networks, including visual and somatomotor regions, show the highest overlap with adults, whereas control and attention networks, such as the FPN, DAN, SAL, exhibit relatively lower correspondence. This pattern is consistent with prior developmental literature indicating earlier maturation and greater cross-individual consistency of sensorimotor systems, whereas control and attention-related systems demonstrate more protracted development and individual variability throughout childhood and adolescence (Sydnor et al., 2021; Tooley et al., 2022). These findings demonstrate that while the overall structure of large-scale functional networks is similar across age groups, network-specific differences remain evident.

Although spatial overlap and community structure indicate broad correspondence in large-scale network organization, the graph theoretic metric participation coefficient (PC) reveals additional nuance in how functional systems interact. PC captures the extent to which regions distribute their connections across multiple networks, providing a complementary view of functional organization beyond spatial topography. Using this metric, we observed more pronounced differences between children and adults than were apparent from similarity measures alone, with adults exhibiting higher PC in dorsal attention, visual, and somatomotor networks. Elevated PC in these systems suggest that, in adults, attentional, sensory, and motor regions participate more broadly in cross-network communication, potentially reflecting greater integration with higher-order association and control systems (Power et al., 2011b; Cole et al., 2013). For example, increased PC in the DAN may reflect more flexible coordination between attentional control processes and other functional networks, consistent with its role in goal-oriented attention and adaptive control (Corbetta and Shulman, 2002; Petersen and Posner, 2012). Similarly, higher PC in visual and somatomotor networks may indicate more distributed interactions supporting efficient sensorimotor integration and perceptual-motor coupling, processes that are known to become more integrated with association networks across development (Marek et al., 2015b).

In contrast, lower PC in these networks in children may reflect a more modular organization, with sensory and motor systems remaining relatively segregated from higher-order networks. While these differences are consistent with developmental theories of network integration, PC is sensitive to how well predefined network boundaries align with the underlying connectivity topography. Consequently, differences in PC between adults and children may reflect variation in parcellation-network correspondence rather than (or in addition to) developmental differences in cross-network integration. Importantly, these differences should be interpreted as descriptive features of network architecture rather than definitive markers of developmental progression, as PC is sensitive to network definition, parcellation, and cross-dataset differences in acquisition and preprocessing (Hallquist and Hillary, 2019). This observed pattern might suggest that developmental differences are more evident in patterns of network interactions than in their spatial organization alone.

This project is marked by several strengths, including a large sample size, the use of demographically matched split-halves for replication, and comparative analyses with adult functional networks. Nonetheless, several limitations should be acknowledged. First, while we assessed reproducibility through split-half analyses, we did not implement more complex validation approaches such as k-fold cross-validation, which may offer additional insights into the stability of network estimates. Our decision to use split-halves was to maintain demographic balance between subsamples. Future studies could incorporate multiple validation strategies to assess variability in network estimates further.

Second, developmental comparisons between ABCD child networks and HCP adult networks must be interpreted in the context of differences in data acquisition parameters and study design. HCP data were collected at a single site and MRI scanner, while ABCD data were collected across 21 sites on different MRI scanners, and HCP and ABCD scan parameters were not harmonized with each other. Such methodological differences can influence FC estimates independent of biological differences through variation in signal-to-noise ratio, motion sensitivity, and scanner effects (Noble et al., 2017; Marek et al., 2019a). Another important methodological consideration is the potential effect of head motion. Developmental differences in head motion are well documented, with children typically exhibiting higher motion than adults. We implemented processing methods (namely motion censoring and global signal regression) previously shown to improve the reliability of RSFC estimates and to best reduce motion-related bias, particularly in group comparisons (Power et al., 2014; Ciric et al., 2017; Satterthwaite et al., 2017). Thus, while motion-related effects were mitigated as much as possible, residual differences in motion or in data loss due to censoring may still contribute to group differences. Nevertheless, we leveraged these large-scale datasets to construct carefully matched child and adult samples (*n* = 1,000 per group) to enable the most rigorous population-level comparison currently feasible. Thus, the reduced similarity between children and adults likely reflects a combination of true developmental effects and methodological differences that cannot be disentangled with the current datasets. Cross-age comparisons should also be interpreted in the context of potential anatomical normalization differences, as commonly used anatomical templates were originally developed using adult samples. However, prior validation work indicates that surface-based registration can accurately align cortical landmarks in children (Ghosh et al., 2010), and that anatomical scaling differences at this developmental stage are relatively modest (Bethlehem et al., 2022). Accordingly, such effects are unlikely to substantially influence the population-level network estimates or broad cross-age patterns reported here.

Third, given the poor signal in the subcortex in the HCP dataset, we were unable to compare groups in subcortical functional network metrics (Seitzman et al., 2020). Moreover, multiband EPI sequences can reduce temporal signal-to-noise ratio in subcortical structures (Todd et al., 2017; Demetriou et al., 2018; Risk et al., 2021), which may influence the reliability of our cortico-subcortical network assignments in the ABCD samples. With continued advancements in scanning parameters and open science efforts making larger datasets more accessible, future work would benefit from making direct comparisons involving the whole brain and will be important for isolating biological from methodological sources of variability.

Fourth, interpretation of PC depends in part on how network boundaries are defined. In the present study, we applied a common parcellation across children and adults to enable direct comparison of cross-network integration within the same reference framework. However, since the Gordon 333 parcels were derived from adults, differences in PC may reflect both developmental variation and parcel boundary variation. While child-tailored parcellations could provide complementary insight, this approach would complicate group comparisons as differences in PC could then also reflect differences in the parcellation schemes. Moreover, emerging developmental work suggests that adult-derived parcellations yield broadly similar conclusions for group-level analyses in children (Tu et al., 2025a). As such, the present findings are best interpreted as reflecting broad differences in network organization, while specific network-level effects should be interpreted with caution. Future work using individual-specific functional mapping or longitudinal designs may provide greater sensitivity for detecting developmental changes in cross-network integration.

Finally, while our findings provide robust estimates of population-specific, group-level functional network organization, this approach necessarily emphasizes central tendencies rather than individual differences. A growing body of research demonstrates variability in network topology across individual children, especially in higher-order association regions (Cui et al., 2020; Keller et al., 2023; Dworetsky et al., 2024; Demeter et al., 2025). Group-averaged parcellations, while useful for identifying central tendencies and supporting the detection of small, population-level developmental effects, inherently smooth over this heterogeneity. As a result, applying group-derived network partitions to examine individual-level brain-behavior relationships may reduce sensitivity to subtle or idiosyncratic features that are functionally important. In this context, population-level and individual-level approaches should be viewed as complementary methods to further our understanding of brain development. Large-sample studies such as the present work provide stable reference frameworks against which individual-specific network configurations can be interpreted. Future work employing individualized network mapping may better capture the functional relevance of network organization to behavioral phenotypes and psychopathology (Gratton et al., 2020; Demeter and Greene, 2024).

## 5 Conclusion

This study provides a large-scale, population-level characterization of functional brain network organization in late childhood and situates this in relation to adult functional network architecture. This benchmark may be useful for detecting atypical patterns of network organization that may underlie emerging cognitive, emotional, or behavioral difficulties, as observed in conditions such as attention-deficit/hyperactivity disorder, autism spectrum disorder, mood disorders, and psychotic-spectrum disorders. By quantifying similarity between child and adult network architecture, this work advances our understanding of which aspects of large-scale organization are broadly stable by age 9–10 years and which are still emerging. Furthermore, this framework provides a foundation for future work integrating individualized brain network profiles with behavioral, cognitive, and clinical measures to better understand developmental trajectories.

## Data Availability

The original contributions presented in the study are included in the article/supplementary material, further inquiries can be directed to the corresponding author.
